# Case Report: Therapeutic Drug Monitoring of Polymyxin B During Continuous Renal Replacement Therapy in Two Pediatric Patients: Do Not Underestimate Extracorporeal Clearance

**DOI:** 10.3389/fphar.2022.822981

**Published:** 2022-03-24

**Authors:** Caifang Xu, Xiaofen Liu, Yun Cui, Xiaolan Huang, Yu Wang, Yaxin Fan, Hailan Wu, Xin Li, Beining Guo, Jing Zhang, Yucai Zhang

**Affiliations:** ^1^ Department of Critical Care Medicine, Shanghai Children’s Hospital, Shanghai Jiao Tong University School of Medicine, Shanghai, China; ^2^ Institute of Antibiotics, Huashan Hospital, Fudan University, Shanghai, China; ^3^ Key Laboratory of Clinical Pharmacology of Antibiotics, National Health Commission of the People’s Republic of China, Shanghai, China; ^4^ Phase I Clinical Trial Center, Huashan Hospital, Fudan University, Shanghai, China

**Keywords:** polymyxin B, CRRT, children, therapeutic drug monitoring, pharmacokinetic

## Abstract

**Background:** Polymyxin B has become the last choice for patient with carbapenem-resistant bacterial infection. However, the optimal dosing of polymyxin B in critically ill children receiving continuous renal replacement therapy (CRRT) remains unclear.

**Case Presentation:** Two cases of critically ill pediatric patients (7 years old) with acute kidney injury requiring continuous renal replacement (CRRT) received polymyxin B treatment due to carbapenem-resistant organism bloodstream infections. Therapeutic drug monitoring (TDM) of polymyxin B was carried out by liquid chromatography tandem mass spectrometry (LC-MS/MS). The average steady-state plasma concentration (C_ss,avg_) of 2–4 mg/L was set as the target level. Initial polymyxin B dose was 1 mg/kg every 12 h, and the C_ss,avg_ at 4–5th dosing were 1.76 and 1.06 mg/L for patient 1 and patient 2, respectively. TDM-guided polymyxin B dose was escalated to 2 mg/kg every 12 h for both patients, resulting in the C_ss,avg_ of 2.60 and 1.73 mg/L, and the infection was controlled subsequently. C_ss,avg_ of polymyxin B with the same dosing regimens and infusion length were different during CRRT and after termination of CRRT for both patients (2.60 mg/L vs. 4.94 mg/L with 2 mg/kg every 12 h in 2 h infusion for patient 1; and 1.73 mg/L vs. 3.53 mg/L with 2 mg/kg every 12 h in 2 h infusion for patient 2). The estimation of drug exposure (estimated by AUC_ss,12h_ at the same dose) during CRRT and cessation of CRRT showed that 45% and 51% of polymyxin B was cleared during CRRT.

**Conclusion:** Our study showed high clearance of polymyxin B through CRRT, and supplanted dosing of polymyxin B is necessary in pediatric patients undergoing CRRT.

## Introduction

Carbapenem-resistant organisms (CROs) are global public health threat due to limited options of antimicrobials. Polymyxins are considered as a last option against CROs (Satlin et al., 2020). The two forms of polymyxins, polymyxin B and colistin, are clinically available. Notwithstanding the similar structures and antimicrobial activity, polymyxin B has some advantages such as direct administration in the active form and lower risk of nephrotoxicity (Tsuji et al., 2019). Furthermore, polymyxin B is the only available form of polymyxins in some countries, although colistin has been more broadly used than polymyxin B internationally (Satlin et al., 2020).

Pharmacokinetic (PK) studies are of paramount importance for suitable dosage of drugs, and to date, research on PK of polymyxin B is conducted mainly with adults (Sandri et al., 2013; Liu et al., 2021). International consensus guidelines recommended a target average steady-state plasma concentration (C_ss,avg_) of 2–4 mg/L for the optimal use of polymyxin B. However, the guidelines have not provided the recommend dosage regimens in children (Tsuji et al., 2019). There are only a few studies that reported polymyxin B used in pediatric patients, with a dose of 1∼4 mg/kg per day (Shih and Gaik, 2014; Gothwal et al., 2016). The huge diversity of doses provided in these studies has made it hard to establish the optimal dose for pediatrics. Furthermore, continuous renal replacement therapy (CRRT) has been increasingly used in critically ill patients with fluid overload or acute kidney injury (AKI). Antibiotics dosage in patients with normal renal function cannot be directly applied to patients under CRRT because of the different pharmacokinetics (Willems et al., 2021). Clinical data also suggested that dose in patients undergoing CRRT could potentially lead to drug underexposure, resulting poor outcomes (Li et al., 2020). Nevertheless, there was a lack of comprehensive guidelines providing antibiotics strategies of polymyxin B for patients undergoing CRRT (Pistolesi et al., 2019). Two reports including three cases of adult patients receiving polymyxin B while CRRT was being operated suggested both continuous venovenous hemodialysis (CVVHD) and continuous venovenous hemofiltration (CVVHF) had a limited impact (no more than 12% clearance) on the removal of polymyxin B (Teng et al., 2008; Sandri et al., 2012). Contrarily, another study with three critical ill adults reported that 45% of colistin was removed during CRRT under the CVVHDF mode (Liu et al., 2020). Whether continuous venovenous hemodiafiltration (CVVHDF) would affect the clearance of polymyxin B in pediatric patients has not been reported. This report evaluates the extent of polymyxin B clearance by continuous hemodiafiltration in two critically ill pediatric patients.

## Case Description

### Patients

Case 1 was a 7-year-old female with a previous history of nephrotic syndrome. She was admitted to the nephrology department due to repeated puffiness for nearly 3 months, on January 23rd, 2021. Two weeks later, she was transferred to the pediatric intensive unit (PICU) for severe pneumocystis pneumonia, respiratory failure, sepsis, and AKI. She was received mechanical ventilation, sulfamethoxazole–trimethoprim, caspofungin, and CRRT. After 3 weeks, carbapenem-resistant *K. Pneumoniae* was isolated from both her urinary catheter and blood. Minimum inhibitory concentrations (MICs) and disk diffusion tests (DISK) of *K. pneumoniae* showed it was only susceptible to polymyxin B (15 mm of DISK and 0.5 mg/L of MIC) and tigecycline (2 mg/L of MIC). Tigecycline combined with fosfomycin was applied, but the efficacy was very limited. Therefore, tigecycline and fosfomycin were replaced by polymyxin B from March 5th 2021 (1 mg/kg every 12 h). On March 18th, due to the recovery of kidney function, CRRT ceased and polymyxin B therapy continued until April 5th.

Case 2 was a 7-year-old female with fulminant myocarditis. She was admitted to the PICU and given venoarterial extracorporeal membrane oxygenation (VA-ECMO) on June 30th 2021. The duration of extracorporeal cardiopulmonary resuscitation (ECPR) was about 70 min, leading to multiple organ dysfunction syndrome (MODS). In addition, CRRT was required (from June 30th to July 25th) for AKI. After 8 days, ECMO was weaned. However, the body temperature elevated between 38 and 40°C, accompanied by an increase in C-reactive protein (123 mg/L) and procalcitonin (16.95 ng/mL). *Stenotrophomonas maltophilia* (multidrug-resistant bacteria, susceptible to cefoperazone–sulbactam and polymyxin B) was identified by blood culture, abdominal fluid and in ECMO catheters. Meanwhile, *Aspergillus fumigatus* was cultured using bronchoalveolar lavage fluid. With cefoperazone–sulbactam combined with linezolid and voriconazole treatment, the clinical symptoms did not ameliorate. Therefore, polymyxin B (17 mm of DISK) was added from July 10th 2021 to August 4th 2021.

### Continuous Renal Replacement Therapy Mode and Parameters

Two patients received CRRT with the CVVHDF mode (GAMBRO, Prismaflex, hemofilter M60). As the coagulation dysfunction, citric acid was used for anticoagulation for both the patients. For patient 1, 25 kg, the settings of CRRT were 30 mL/min for blood flow, 60 mL/h for citric acid, 100 mL/h for replacement fluid, 300 mL/h for dialysate fluid, and 60 mL/h for effluent. For patient 2, 20 kg, 40 mL/min for blood flow, 64 mL/h for citric acid, 300 mL/h for replacement fluid, 150 mL/h for dialysate fluid, and 80 mL/h for effluent was set.

### Samples and Therapeutic Drug Monitoring

For both patients, trough and peak blood samples at steady state (about 0.5 h before and after the infusion of 4–5th dosing) were collected for TDM of polymyxin B. If the dosage regimens needed to be adjusted according to TDM, trough and peak blood samples after the third post-adjustment dose were collected. In the meantime, post-filtration blood samples were collected at the same time to evaluate the drug elimination during CRRT.

Polymyxins B1 and B2 were determined following a validated liquid chromatography tandem with mass spectrometry (LC-MS/MS) method (Liu et al., 2020). In brief, plasma samples were mixed with internal standard (polymyxin E2) and 30% ammonia and then loaded on a preconditioned (conditioned by methanol and water as per product instruction) 96-well Oasis WCX plate (30 mg, Waters Corporation, Milford, MA, United States). The WCX plate was washed and eluted by 30% acetonitrile in water (containing 6% formic acid, v/v). The elute was injected into LC-MS/MS (Shimadzu 30A series coupled with AB Sciex Triple Quad 5500 mass spectrometry platform) for analysis. Concentrations of polymyxin B were calculated by the sum of polymyxins B1 and B2 by the molar terms and molecular weight. The C_ss,avg_ of 2–4 mg/L (estimated by the average of peak and trough concentration) of polymyxin B was set as the target concentration.

### AUC Estimates

Compartmental modeling using WinNonlin 8.0 was applied for PK parameters estimation and AUC calculation. Due to the limited data with C_max_ and C_trough_ for the second patient, an alternative equation-based approach for two PK samples methods was used for the AUC_24h_ estimates (Pai et al., 2014) under similar eGFR. The equations are given as follows:

$$K_{e}=\frac{LnC_{max}-\mathrm{Ln}C_{trough}}{t_{2}-t_{infusion}},$$

$$AUC_{24h}=\left(\frac{t_{infusion}\times C_{max}+C_{trough}}{2}+\frac{C_{max}-C_{trough}}{k_{e}}\right)\times n,$$

where *K*_*e*_ is the elimination rate constant, *t*_*2*_ is the drug administration interval, *t*_*infusion*_ is the infusion length, and *n* is the administration times during 24 h.

## Results

### Polymyxin B and Plasma Concentrations

**FIGURE 1:**
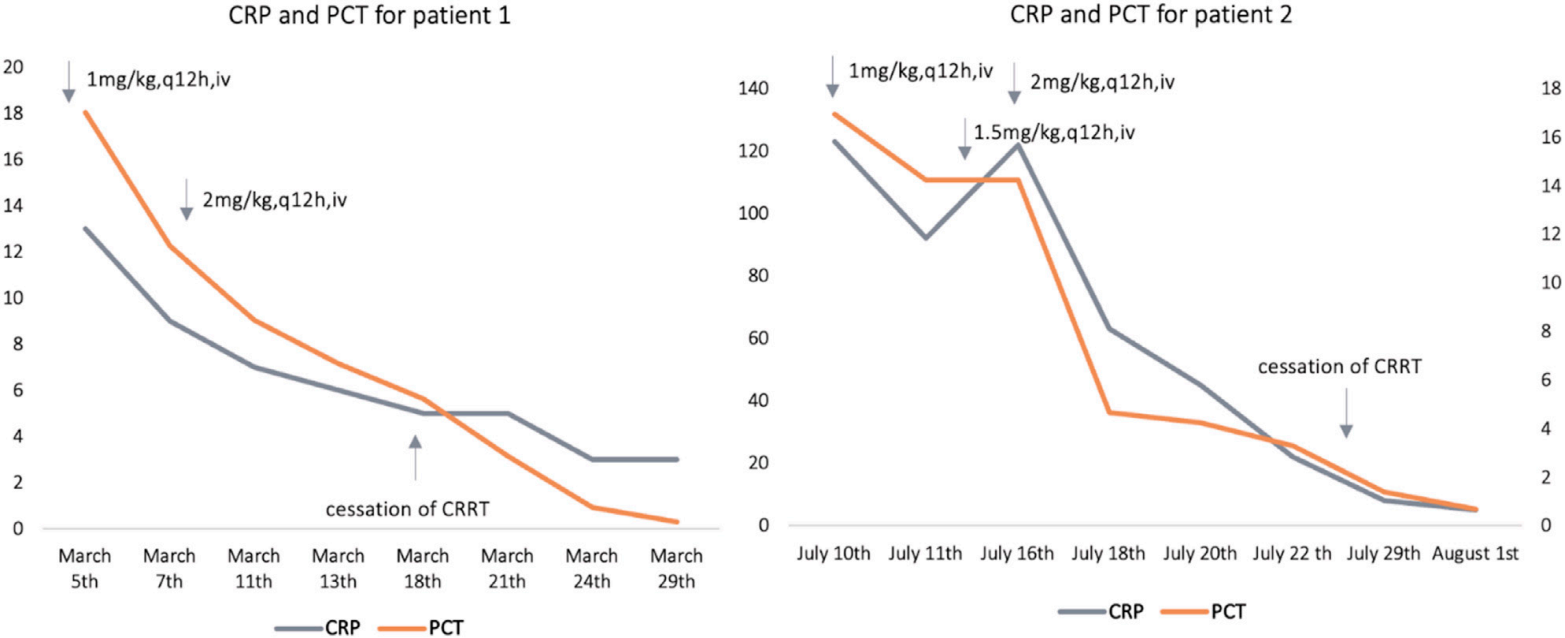
Polymyxin B dose regimens with the concomitant drugs and estimated C-reactive protein (CRP), and procalcitonin (PCT) levels at different time points; for patient 1, cessation of CRRT (continuous renal replacement) was on 18^th^ March; for patient 2, cessation of CRRT was on 25^th^ July. CRP: C-reactive protein (mg/L), PCT: procalcitonin (ng/mL).

After treatment with polymyxin B (1 mg/kg, every 12 h, C_ss,avg_ = 1.76 mg/L), C-reactive protein (CRP) of patient 1 decreased from 13 mg/L to 5 mg/L, and procalcitonin (PCT) fell from 18.04 ng/mL to 5.62 ng/mL. Nonetheless, the body temperature of patient 1 fluctuated around 38.0°C. When C_ss,avg_ ascended to 2.60 mg/L, CRP and PCT decreased to the normal range (CRP ≤ 5 mg/L, PCT 0.02 ng/ml) and the body temperature to normal. For patient 2, the levels of CRP and PCT did not decline until the C_ss,avg_ of polymyxin B reached 1.73 mg/L. The concentrations of polymyxin B at corresponding dosing regimens for the two patients and estimated glomerular filtration rate (eGFR, calculated by Schwartz formula) levels are presented in Table 1. In addition, the polymyxin B dose regimens with the concomitant drugs and C-reactive protein (CRP) and procalcitonin (PCT) levels at different times are shown in Figure 1.

**TABLE 1:** Concentrations at different dosing regimens.

Patient No.	Polymyxin B dosing regimens (q12 h)	CRRT	eGFR* (mL/min)	Peak (mg/L)	Trough (mg/L)	Average (mg/L)
1	1 mg/kg with 1 h infusion	Yes	69.7	3.31	0.20	1.76
	2 mg/kg with 2 h infusion	Yes	111.3	4.79	0.41	2.60
	2 mg/kg with 2 h infusion	No	108.7	7.67	2.21	4.94
	1.5 mg/kg with 2 h infusion and 0.5 mg/kg inhalation	No	101.6	6.95	3.39	5.17
	1 mg/kg with 2 h infusion	No	116.8	6.45	0.80	2.63
2	1 mg/kg with 2 h infusion	Yes	40.2	1.87	0.23	1.06
	1.5 mg/kg with 2 h infusion	Yes	71.8	1.70	0.22	0.96
	2 mg/kg with 2 h infusion	Yes	60.8	3.19	0.26	1.73
	2 mg/kg with 2 h infusion	No	128.9	5.24	1.82	3.53

Comparing C_ss,avg_ of polymyxin B with the same dosing regimens and infusion length, it showed significantly lower concentrations during CRRT than that after termination of CRRT for both patients (2.60 mg/L vs. 4.94 mg/L with 2 mg/kg every 12 h in 2 h infusion for patient 1; and 1.73 mg/L vs. 3.53 mg/L with 2 mg/kg every 12 h in 2 h infusion for patient 2).

### Drug Removal by the Hemofilter Membrane

**FIGURE 2:**
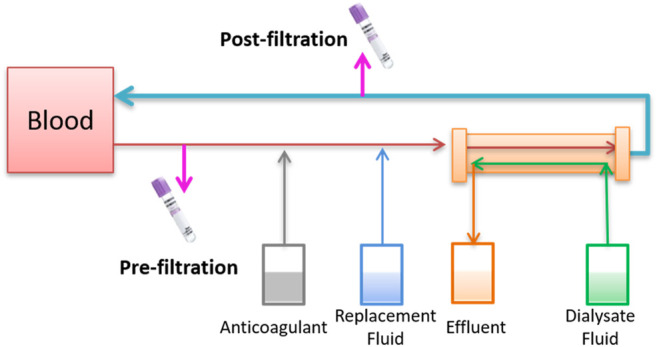
Schematic diagram of blood collection in the extracorporeal catheter prefilter and postfilter of CRRT.

To assess whether CRRT could remove polymyxin B, blood samples from prefilter and postfilter were measured (Figure 2). The same type of hemofilter membrane was used for both patients. The concentrations of post-filtration and pre-filtration blood samples were 0.23 and 3.31 mg/L, respectively, which indicated 93.1% of polymyxin B was removed during the CVVHDF mode for patient 1. Two more pairs of pre- and post-filtration blood samples were evaluated for the drug removal during CRRT, and plasma concentrations showed 90.9–95.3% of polymyxin B was cleared. Similarly, 86.6% of polymyxin B was cleared for patient 2, with the concentrations of 0.43 and 3.19 mg/L (Table 2).

**TABLE 2:** Concentration of polymyxin B of prefilter and postfilter at 0.5 h after infusion during CRRT.

Patient No.	Pre-filtration plasma concentration (mg/L)	Post-filtration plasma concentration (mg/L)	Ratio of drug loss (%)
1	3.31	0.23	93.1
	4.02	0.19	95.3
	5.55	0.50	90.9
2	3.19	0.43	86.6

**FIGURE 3:**
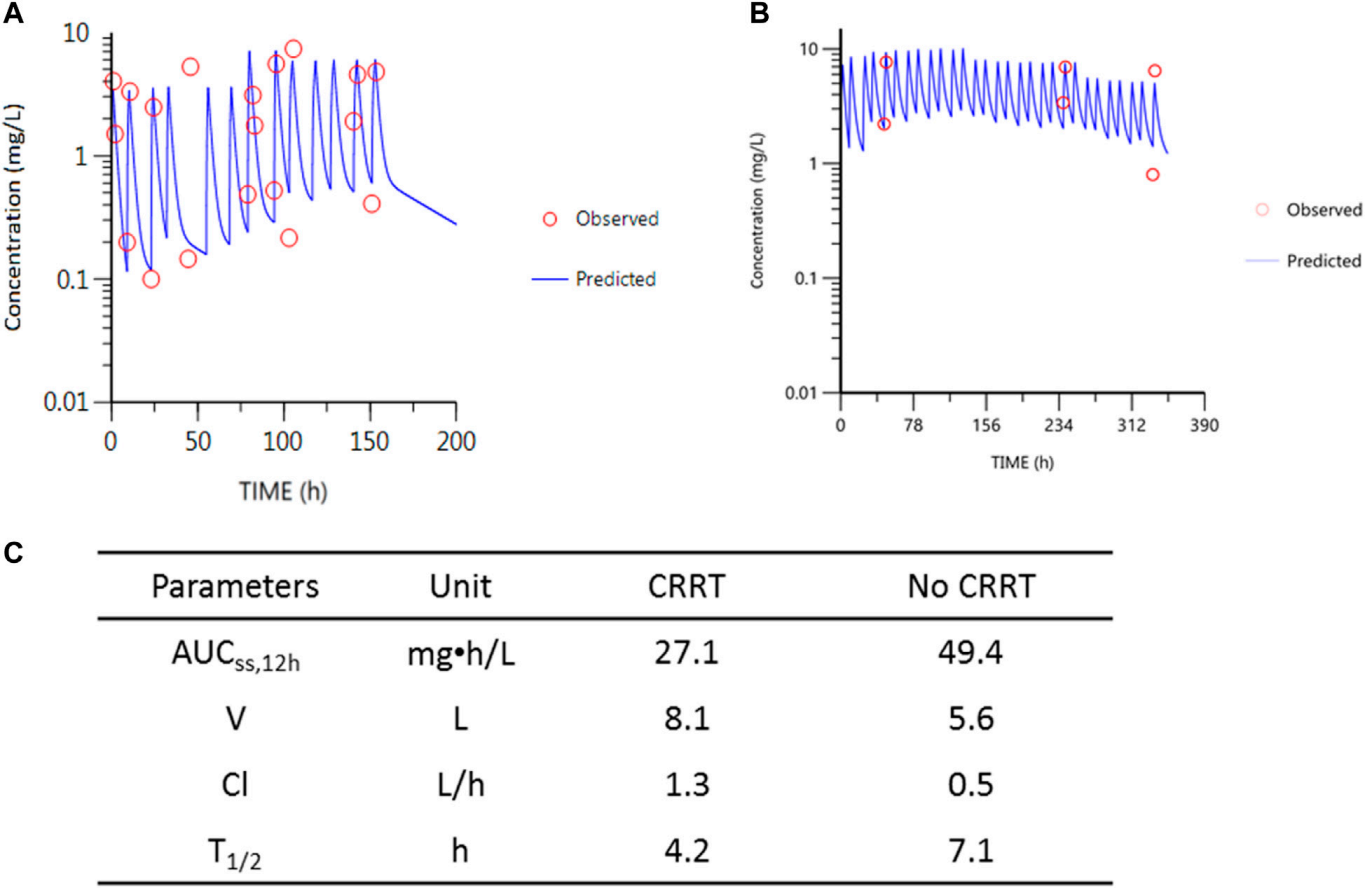
PK parameters of polymyxin B and the simulations for patient 1. **(A)** Observed concentrations vs. predicted concentrations during CRRT. **(B)** Observed concentrations vs. predicted concentrations after recess of CRRT. The last two samples in **(B)** were deviated from the blue line due to the dose adjustments from 1.5 mg/kg (with 0.5 mg/kg inhalation) to 1.0 mg/kg infusion. Red circles represent observed concentrations; blue line represents the predicted concentrations according to the two-compartmental PK model. **(C)** Table for the PK parameters during CRRT and without CRRT.

Compartmental modeling was conducted using WinNonlin 8.0 for patient 1 whose samples were collected during and recess of CRRT. Drug exposure (estimated by AUC_ss,12h_ at the same dose) during CRRT and recess of CRRT through a preliminary pharmacokinetic modeling for patient 1 was estimated. The results showed that 45% of polymyxin B was removed during CRRT (Figure 3). By applying the equation-based method for calculating AUC_24h_ for the first patient, 57% of polymyxin B was removed during CRRT, which was similar to that obtained using the compartmental modeling method. The same equation-based method was applied for the AUC_24h_ estimation of the second patient. It was calculated that AUC_24h_ was 30.2 mg•h/L and 60.6 mg•h/L for the 2 g and 2 h infusion of polymyxin B during CRRT and without CRRT, respectively, indicating 51% of polymyxin B lost during CRRT.

## Discussion

The incidence of carbapenem-resistant organism infection is increasing globally, mainly carbapenem-resistant Enterobacteriaceae and *Acinetobacter baumannii*. Polymyxin is considered as the salvage therapy for aforementioned infections. The international consensus guideline recommended a polymyxin B dose of 1.25–1.5 mg/kg every 12 h for patients with severe infection patients. With doses of 2.5 and 3.0 mg/kg/day, 90% of adults would be expected to achieve a C_ss,avg_ of 1.8 and 2.2 mg/L, respectively (Tsuji et al., 2019). In addition, the consensus guideline suggested that polymyxin B dose need not be adjusted during CRRT. However, the guideline has not provided the recommended dosage regimens for children. In our study, to achieve the target C_ss,avg_, a polymyxin B dose of 4.0 mg/kg/d was needed, which was far from the recommended doses in adults.

Young children show changes in drug absorption, disposition, metabolism, and excretion of drugs (Kearns et al., 2003; Downes et al., 2014). For example, in absorptive surfaces such as the gastrointestinal tract, skin, and pulmonary tree, it can influence the rate and extent of the bioavailability of drugs. Age-related changes in body composition alter the volume of distribution of antibiotics. Delayed maturation of drug-metabolizing enzyme activity and developmental changes in renal function account for the difference in drug clearance. Additionally, in critically ill patients, the hemodynamic alterations or renal dysfunction during critical illness pathophysiologically change the volume of distribution, protein binding, and drug clearance. In PICU patients, antibiotic concentrations are outside of the therapeutic window up to 95%, while these non-target concentrations in adult critically ill patients are up to 41% (Hartman et al., 2019). In our study, the clearance of polymyxin B (Cl) was 1.3 L/h (correspondingly 0.05 L/h/kg) during CRRT and 0.5 L/h (correspondingly 0.02 L/h/kg) without CRRT, which was in the range of Cl reported previously (0.02–0.07 L/h/kg in critically ill patients) (Sandri et al., 2013; Liu et al., 2021). The half-life (t_1/2_) of polymyxin B was different from that during CRRT and without CRRT (4.2 vs. 7.1 h), but in line with previous reports (healthy subjects (∼5 h) and critically ill patients (∼11.9 h) (Sandri et al., 2013; Liu et al., 2021). These suggested that changes in the dose of polymyxin B in these two patients had little to do with the special population of children themselves.

On the other hand, antibiotics with large molecular weight and high protein binding rate forming drug–protein complexes are not easily removed by CRRT (Pistolesi et al., 2019). Therefore, in theory, polymyxin B is difficult to be removed during CRRT because of its large molecular weight of more than 1,000 Da (e.g., 1,203, 1,189, and 1,203 for polymyxins B1, B2, and B1-Ile, respectively) and protein binding rate of 58% (Sandri et al., 2013). Two reports with three cases of adult patients suggested that only 5.6% and 12.2% of polymyxin B were removed by CVVHD and 5.0% was removed by CVVHF (Teng et al., 2008; Sandri et al., 2012). Conversely, another study with three critically ill adults reported that 45% of colistin was removed during CRRT under the CVVHDF mode (Nikolaos et al., 2012). In this study, both cases showed significant drug clearance of polymyxin B during CRRT with the CVVHDF mode. Comparing C_ss,avg_ of polymyxin B with the same dosing regimens and infusion length, it showed significantly lower concentrations during CRRT than that after termination of CRRT for both patients (2.60 mg/L vs. 4.94 mg/L with 2 mg/kg every 12 h in 2 h infusion for patient 1; and 1.73 mg/L vs. 3.53 mg/L with 2 mg/kg every 12 h in 2 h infusion for patient 2). This was also confirmed by the estimation of drug exposure (estimated by AUC_ss,12h_ at the same dose) during CRRT and recess of CRRT through a preliminary pharmacokinetic. Results showed that 45% and 51% of polymyxin B was clearance during CRRT which was much higher than that in the reported studies (no more than 12%) (Teng et al., 2008; Sandri et al., 2012). In addition, the plasma concentrations in prefilter and postfilter membranes showed 86.6–95.3% drug removal during filtration, suggesting the remarkable extracorporeal clearance. A previous study reported colistin absorption by the hemofilter, and the removal of dialysis contributed to its extracorporeal clearance (Nikolaos et al., 2012). In our study, we measured the concentration of the effluent fluid of CRRT, but due to the dilution effect and the large volume of the effluent (more than 5 L for 12 h), the concentration of polymyxin B was lower than the detection limit of the LC-MS/MS assay. Further studies are needed to evaluate the drug removal by adsorption of the hemofilter. Since this study only provided two cases, the exact clearance of polymyxin B through CRRT indicating supplanted dosing is necessary for pediatric patients undergoing CRRT.

## Conclusion

CRRT (CVVHDF mode) significantly affects the drug concentrations and exposure of polymyxin B in the present two cases, indicating supplantation of polymyxin B is necessary in pediatrics undergoing CRRT. The TDM of polymyxin B is critically important for the dosing regimen optimization for patients undergoing CRRT. Larger PK studies are urgently needed to further refine dosing recommendations of polymyxin B in pediatric population receiving CRRT.

## Data Availability

The original contributions presented in the study are included in the article/Supplementary Material, further inquiries can be directed to the corresponding authors.
